# The effectiveness of penile curvature treatment by cavernous body rotation and plication of the tunica albuginea

**DOI:** 10.1186/s12610-023-00186-y

**Published:** 2023-03-30

**Authors:** Vladimir Vorobev, Vladimir Beloborodov, Andrey Sherbatykh, Alexey Kalyagin, Olga Baklanova, Sergei Popov, Stepan Sidorov

**Affiliations:** 1 Department of General Surgery, Irkutsk State Medical University, Irkutsk, Russian Federation; 2 Department of Theoretical Surgery, Irkutsk State Medical University, Irkutsk, Russian Federation; 3 Department of Introduction to Internal Medicine, Irkutsk State Medical University, Irkutsk, Russian Federation; 4 Department of Urology, GBUZ State Oncology Hospital, Irkutsk, Russian Federation; 5 Department of Urology, Irkutsk State Medical University, Irkutsk, Russian Federation

**Keywords:** Penile curvature, Congenital curvature of the penis, Corporoplasty, Rotational corporoplasty, Plication of the tunica albuginea of the corpora cavernosa, Corporal rotation, Courbure pénienne, Courbure congénitale du Pénis, Corporoplastie, Corporoplastie rotationnelle, Plicature de la Tunique albuginée des Corps caverneux, Rotation corporelle

## Abstract

**Background:**

There are several approaches to the surgical treatment of the penile curvature conditionally divided into three large groups: tunica albuginea plication (TAP), corpus cavernosum rotation (CR), and transplantation of various materials. The study aims to compare the effectiveness of TAP and CR techniques in the treatment of penile curvature. There was a prospective randomized study of the effectiveness of surgical treatment of patients with an established diagnosis of the penile curvature from 2017 to 2020 in Irkutsk, Russian Federation. The final analysis of the results included 22 cases.

**Results:**

The analysis of the comparative intergroup effectiveness of the treatment performed based on the criteria established in the study showed good treatment results in 8 (88.8%) patients in the CR group and 9 (69.2%) patients in the TAP group (p = 0.577). The other patients obtained satisfactory results. There were no negative outcomes. Simple logistic regression analysis showed that the preoperative flexion angle > 60 degrees (OR 2.7; 95% CI 0.12; 5.28; p = 0.040) was significant in predicting the complaints of penile shortening during TAP. Both methods are safe, effective, and bring minimal risk of complications.

**Conclusion:**

Thus, the effectiveness of both treatment methods is comparable. However, TAP surgery is not recommended for patients with an initial curvature of more than 60 degrees.

## Résumé

**Contexte:**

Il existe plusieurs approches du traitement chirurgical de la courbure pénienne, divisées en trois grands groupes : plicature de la tunique albuginée (PTA), rotation du corps caverneux (RCC) et transplantation de divers matériaux. La présente étude vise à comparer l’efficacité des techniques PTA et RCC dans le traitement de la courbure du pénis. Il s’agit d’une étude prospective randomisée de l’efficacité du traitement chirurgical de patients ayant un diagnostic établi de courbure du pénis, de 2017 à 2020 à Irkoutsk, en Fédération de Russie. L’analyse finale des résultats comprend 22 cas.

**Résultats:**

L’analyse de l’efficacité comparative intergroupe du traitement effectué sur la base des critères établis dans l’étude, a montré de bons résultats du traitement chez 8 (88,8%) patients du groupe RCC et 9 (69,2%) patients du groupe PTA (*p* = 0,577). Les autres patients ont obtenu des résultats satisfaisants. Il n’y a eu aucun résultat négatif. Une analyse de régression logistique simple a montré qu’un angle de flexion préopératoire > 60 degrés (OR 2,7 ; IC à 95 % 0,12 ; 5,28 ; *p* = 0,040) était significatif pour prédire les plaintes de raccourcissement du pénis avec l’approche de la PTA. Les deux méthodes sont sûres, efficaces et présentent un risque minimal de complications.

**Conclusions:**

Ainsi, l’efficacité des deux méthodes de traitement est comparable. Cependant, la chirurgie avec l’approche de la PTA n’est pas recommandée chez les patients ayant une courbure initiale de plus de 60 degrés.

## Background

Penile curvature due to various reasons (congenital or acquired) is a disease leading to persistent impairment of copulatory function. The epidemiology is definitively unclear as there are not enough large-scale researches available. The most famous work by Yachia et al. [1] describes a 0.6% chance of birth a boy with penile curvature. There is also information about a higher incidence of anomalies – 4 to 10%, based on the data of the Birth Defects Monitoring Program (BDMP, USA) and data from other authors [2]. The prevalence of acquired curvature varies from 0.5 to 13% [3]. Many authors do not include curvature less than 20 degrees in the statistics, since it does not affect the copulatory act.

There are four main mechanisms of congenital curvatures development: cutaneous or subcutaneous chord, the disproportion of the corpus cavernosum, or congenitally short urethra [2]. The congenital type is genetic; aberrant expression of COL1A1 and COL6A1 affects tunica dartos abnormalities and the severity of curvature [4].

Based on severity the curvature can be insignificant (less than 20 degrees), mild (20–30 degrees), moderate (30–60 degrees), and severe (more than 60 degrees) [4, 5].

The diagnosis is not difficult and is based on anamnesis and assessing the erect penis (based on photographs of the patient in at least three projections, or with artificial induction of erection in a hospital) [6].

There are several approaches to the surgical treatment of curvature of the penis divided into three large groups: tunica albuginea plication (TAP), corpus cavernosum rotation (CR), and transplantation of various materials. Orthoplasty, as a method of removing chords, is not a complete medical operation for most cases of curvature. The plication technique was first described at the beginning of the 19th century (Philip Syng Physick), but modern TAP methods are based mainly on a modified procedure proposed by Nesbit in 1965 [6].

Interestingly, the analysis of the surgical treatment effectiveness depending on age (prepubertal, average age 8.3 years; postpubertal, average age 16.2 years) demonstrated comparable long-term results (78% and 83% efficacy for the respective groups at 38,7 and 45.1 months), which indicates the possibility of successful treatment at any age [7].

Nowadays there are various modifications of TAP by Nesbit. For example, a comparative study proved the technical superiority of the medial dissection of the neurovascular bundle without excision of the dorsal vein over the lateral variant. Meanwhile, the probability of penile shortening, the effectiveness of its straightening, and postoperative numbness of the glans penis did not differ significantly in both groups [8]. There are also modifications without circumcision, which have a similar high efficiency (96%) and comparable risks of complications [9, 10].

In general, the effectiveness of TAP is about 90–100% in the first postoperative year, typically accompanied by a loss of sensitivity of the glans penis (8.3% in the first year after surgery) and its shortening (26.6–80%; from 1.5 up to 3–5 cm, depending on the bend) [11–15]. Long-term results indicate a 35–48% risk of curvature recurrence, as well as the risk of anxiety about palpable indurations in the plication area [13, 16].

For the theoretical calculation of the degree of penis shortening (L) depending on the curvature angle (Y) and the girth of the erect penis (C), the authors proposed a simple formula (L = CY / 180) and a table-nomogram, which can be useful in planning and discussing TAP operations [17].

The significant disadvantages of TAP, such as a significant shortening of the penis, the likelihood of repeated curvature, decreased sensitivity, and impaired sexual function led to the need to develop alternative surgical techniques.

Snow [18] and Kass [19] proposed the dorsal rotation of the corpora cavernosa [6], and subsequently, Shaeer modified it in three technics: the first [20] and the second [21] include corporotomy, and the third - without it [22].

The rotation technique with corporotomy provides high efficiency (90%) with an average penis shortening of about 5 mm [23]. The modified technique provides high efficiency (close to 100%) with insignificant (less than 1 cm) shortening of the penis, regardless of the bending angle, however, there is a slight decrease of the girth of the erect penis [22, 24, 25].

The study aims to compare the effectiveness of the TAP and CR techniques in the treatment of penile curvature.

## Materials and methods

### Research design

The clinical trial was approved by the local ethics committee (protocol №2, 15.11.2017) of the Irkutsk State Medical University (ISMU). A prospective, double-blind, randomized (by the method of the blocked fixed randomization) study was carried out in a surgical hospital of the Irkutsk City Clinical Hospital No. 1 and Saint Luke hospital.

The clinical part of the study includes an analysis of surgical treatment results of patients with an established diagnosis of penile curvature from 2017 to 2020.

The inclusion criteria: adult men with the congenital or idiopathic penile curvature, ventral / dorsal / lateral, completed IIEF 5 (International Index of Erectile Function). Non-inclusion criteria: Peyronie’s disease, patient failure, complex curvature, curvature less than 20 degrees, erectile disfunctions, moderate or sever, previously performed operations for penile curvature. Exclusion criteria: changes in the surgical team; the patient dropped out of the study at any stage.

The examination included anamnestic (to establish the type of disease, concomitant diseases, etc.), IIEF5 test, clinical blood and urine, cultural urine test, complete biochemical profile of the blood, ultrasound and tomographic of the urinary system and corpus cavernosum.

To clarify the state of the corpora cavernosa and the characteristics of blood flow during erection, an ultrasound examination of the penis with induction of an erection was performed (intracavernous injection of 10 mg of alprostadil into each cavernous body).

The measurement of the length and girth of the erect penis is performed with a flexible measuring tape, or by the “string-ruler” method from the symphysis to the extreme point of the glans penis. The IIFE-5 scale allowed assessing the state of erectile function before surgery and 3 months after surgery. The curvature angle was measured using a protractor.

Upon reaching a month and three months after the surgery, all patients underwent the standard assessment of the state established by the study protocol: consultation with a urologist, clinical blood and urine tests, ultrasound examination of the penis, measurement of its length and curvature. Patient complaints were recorded. A similar examination was repeated every 6–12 months.

At the planning stage of the study, the required sample size was calculated using the STATISTICA software for Windows version 10.0. According to the results of several studies presented in the introduction (averaged CR and TAP efficacy indicators, the risk of penile shortening, changes in its girth, etc.), the calculation showed that 8 (the Chi-square method) and 9 (the t-test method) patients in each of the comparison groups will be enough to reproduce the differences in the postoperative state with the probabilities of error of the first and second types equal to 0.05 and 0.2, respectively. Thus, the required total sample size (two comparison groups of patients) should be at least 18 patients.

**Fig. 1:**
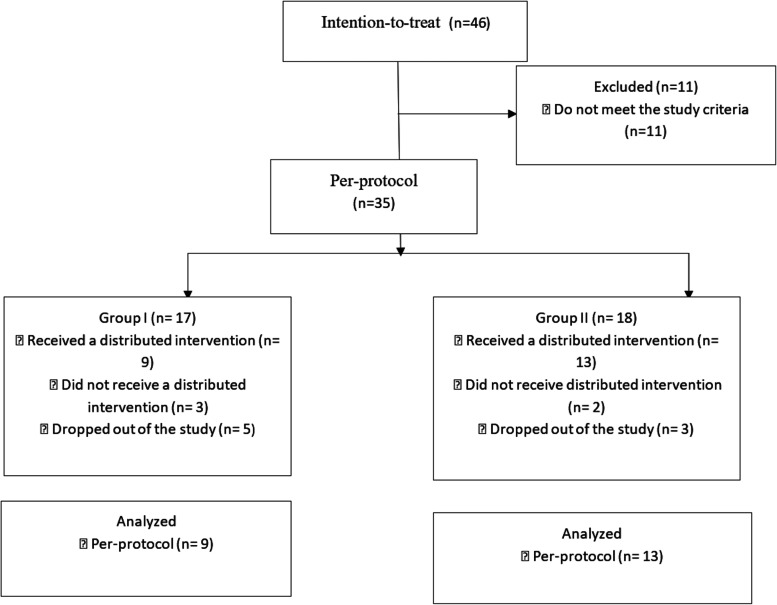
Block diagram of the study design. Note: Intention-to-treat – those who have passed the assessment for acceptability, not included in the study. Per-protocol – included in the study according to the protocol

The recruitment of patients who met the inclusion criteria was carried out prospectively using the continuous sampling method until the desired sample size was reached. During this period, penile curvature was detected in 46 patients. Only 35 patients met the study inclusion criteria. All included patients were randomized into two groups based on the approved study protocol. In both groups, perioperative management was performed according to the fast-track surgery treatment protocol (FTS, approved by the ethical committee of the ISMU). The first group (group I) of patients underwent surgical treatment using Shaeer’s corporal rotation III [20], and the patients of the second group (group II) underwent TAP procedure according to Nesbit in medial modification with or without circumcision Fig. 1.

### Checkpoints

The primary “hard” endpoints were considered: freedom from complications Clavien-Dindo > 1.

The secondary “soft” checkpoints of clinical efficacy were the data of the subsequent postoperative examination: normal IIEF indices, the angle of postoperative curvature of the penis less than 10 degrees, restoration of the sensitivity of the glans penis, and the last control examination at least three months after the operation.

### Statistical analysis

The initial data and surgical treatment results were analyzed using STATISTICA software for Windows version 10.0 (Statsoft, Inc, USA), SPSS Statistics version 23.0 (IBM, USA), and Stata version 16.0 (StataCorp, USA).

The Shapiro-Wilk test helped to verify the hypothesis of the normality of features distribution. The Leuven test allowed verifying the equality of the variances of the distributions of the features.

For descriptive statistics of quantitative normally distributed features with equality of variances, there are parametric methods: calculation of average values and standard deviations. For quantitative features with a distribution other than normal and qualitative ordinal features, there are nonparametric methods: calculating medians and the corresponding interval between the 25th and 75th percentiles (Q1; Q3). For qualitative nominal features, relative frequencies served as a percentage.

To determine the reliability of differences in paired comparisons, there was the nonparametric McNemar test (nominal data groups); the nonparametric test of Wilcoxon signs (ordinal data groups); the paired t-test (groups of continuous data with a normal distribution of the feature); and the nonparametric test of Wilcoxon signs (groups of continuous data with a non-normal distribution of the feature).

To determine the reliability of differences intergroup (independent) comparisons, there was the Chi-square test (nominal data groups), nonparametric Mann-Whitney U-test (ordinal data groups), Student’s test (groups of continuous data with a normal distribution of the feature), nonparametric U-Mann-Whitney test (groups of continuous data with a distribution other than normal).

In case of statistically significant differences in the groups, to overcome the problems of multiple comparisons the nonparametric Mann-Whitney test with Bonferroni correction allowed performing a pairwise comparison of the groups.

Simple and multiple logistic regression helped to identify predictor variables for a binary response variable. The predictor variables were selected according to the initial and postoperative parameters. Cox proportional risks regression helped to assess the correlation between one or more continuous or categorical variables and the time to an adverse event. The significance level for all methods is p ≤ 0.05.

Comparison of the groups is presented by the composite endpoints method [26].

### Treatment protocols

Perioperative preparation was carried out according to the ERAS program. Preoperative patient education and discussion were conducted by a multidisciplinary team. Patients were recommended to stop smoking and alcohol cessation for the entire perioperative period. The patient was allowed to consume liquids and carbohydrate mixtures 2 h before surgery, bowels preparing was not performed – the patient was prescribed a slag-free diet four days before surgery, and premedication of NSAIDs (ketoprofen) was performed. Antibacterial prophylaxis was performed once, 30 min before the operation. The protocol of multimodal anesthesia was applied according to the decision of the team of anesthesiologists.

All operations were performed with the induction of an erection by injecting saline solution into the cavernous bodies. When performing the plication, the standard Nesbitt procedure was used, with modifications in several cases: without deglovation, medial variant. Thus, not all patients underwent penile scalping. Dissection of the neurovascular bundle was performed laterally or medially. After the tunica albuginea was exposed, the necessary volume of plication’s sutures was estimated to eliminate the curvature. Then stitches were performed on the marked zones.

The rotation was carried out according to the Shaeer’s corporate rotation III method, a reference to which was presented earlier. The operation involves the isolation of the dorsal neurovascular bundle and the execution of longitudinal colliding separate sutures throughout the cavernous bodies, which unfold and fix the dorsal surfaces in the direction of each other. Rotary seams are applied until the desired result is achieved.

Intraoperatively, non-absorbable suture material Proline was used on cavernous bodies, monopolar coagulation was not used. The skin suture by the Monocryl 5 − 0, cyanoacrylic glue was applied to the wound. In the postoperative period, on the first day after surgery, only NSAIDs (ketoprofen) were used for pain control, it was allowed to consume liquid after 3 h and solid food 6 h after. Infusion therapy was ended with the resumption of nutrition. The urethra was intraoperatively catheterized, the catheter was removed 6 h later. The next day after the surgery, the patient underwent the final assessment, control blood and urine tests, an ultrasound examination. In the case of a satisfactory condition, the patient was discharged.

## Results

Of the 35 patients initially included in both comparison groups, 13 were subsequently excluded from the study. From the group of patients excluded from the study, 5 dropped out due to deviations from the study protocol, and 8 – for personal reasons. Deviations from the protocol were conventionally divided into primary, secondary, and tertiary (Table 1).

**Table 1:** Analysis of deviations from the research protocol

Deviations	Characteristic	Group I CR (*n* = 17)	Group II TAP (*n* = 18)	*P*
Primary deviations	Different treatment method has been chosen, n (%)	2 (11.7%)	0 (0%)	0.157
No primary deviations from protocol, n		15	18	-
Secondary deviations	Intraoperative change of tactics, n (%)	1 (6.6%)	2 (11.1%)	0.685
No primary and secondary deviations from the protocol, n		14	16	-
Tertiary deviations	Changing the diagnosis, n (%)	0 (0%)	0 (0%)	-
Protocol feasibility, n (%)		14(82.3)	16(88.8)	0.878

To determine the value of P, a Chi-square comparison was used

*CR* corporal rotation, *TAP *tunica albuginea plication

Primary deviations - before the surgical intervention

Secondary deviations - a change in intraoperative tactics

Tertiary deviations – after the surgical intervention

The primary deviation from the protocol meant a forced change in the treatment protocol before the surgical intervention. For two patients of the first group, we had to change the surgical tactics to plasty of the tunica albuginea with the use of graft due to the complex variant of curvature.

Secondary deviations occurred in a single case in the first group and two cases in the second group, due to a change in intraoperative tactics. For the patient of the first group, due to the small bending angle (26 degrees) and his wishes, intraoperatively we assessed the prospect of performing TAP, which was performed. In the second group, a combined TAP correction in combination with buccal graft transplantation was performed intraoperatively due to a significant bending angle and complex bending shape.

There were no tertiary deviations from the study protocol.

The indicator of the likelihood of completing the treatment protocol, considering all three groups of deviations in this sample, was 82.3% and 88.8% for clinical groups I and II (*p* = 0.878), respectively. Due to such forced deviations from the study protocol, these patients were excluded from the analysis according to the protocol (per-protocol) as not meeting the study criteria.

There were 5 (21.7%) patients of group I and 3 (13.0%) of group II who refused to participate in the study at any stage. The completeness of clinical follow-up for patients in both groups was 39.1% (9 cases) for group I and 56.5% (13 cases) for group II. The analysis of the effectiveness of postoperative follow-up demonstrated its comparability in both groups (by the Chi-squared test; *p* = 0.482).

Thus, the final clinical analysis included 22 patients (per-protocol) meeting all the study criteria. Of these, 2 groups of patients were formed. The patients of the CR method (*n* = 9, group I) and the patients of the TAP procedure (*n* = 13, group II).

Table 2 presents comparative data on the initial parameters of patients in the study groups.

**Table 2:** Comparative characteristics of patients in comparison groups before surgery

Parameters	Group I CR (*n* = 9)	Group II TAP (*n* = 13)	Р
**General:**			
Age, years	27.4 (±9.8)	28.2 (±10.27)	0.858
Height, cm	175.7 (±4.4)	174.4 (±8.5)	0.676
Weight, kg	71.7 (±8.4)	71.5 (±12.4)	0.960
**Indicators of penile status:**			
Penile curvature angle, degrees	48.8 (±17.1)	48.2 (±14.9)	0.924
Length of an erect penis, cm	16.7 (±1.9)	16.1 (±2.2)	0.473
The girth of the erect penis, cm	13.3 (±1.6)	12.8 (±1.48)	0.523
Vertical curvature, n (%)	7 (77.7%)	9 (69.2%)	0.218
Congenital curvature, n (%)	7 (77.7%)	9 (69.2%)	0.218
**Anamnesis:**			
Smoking, n (%)	4 (44.4%)	8 (61.5%)	0.664
Alcohol abuse, n (%)	1 (11.1%)	1 (7.6%)	0.802
Allergy, n (%)	2 (22.2%)	3 (23%)	0.970
IIEF5 before surgery, scores	21 (21;22)	20 (20;22)	0.300

To determine the value of P, the Student’s criterion was used (in groups of continuous data with a normal distribution of the trait; described as mean +/-SD;), chi-square criterion (in groups of nominal data; described as n (%)), nonparametric Mann-Whitney U-criterion (in groups of continuous data with a distribution of different from normal; denoted as median and 25–75 quartiles.)

*IIEF5 *International Index of Erectile Function

*CR* corporal rotation, *TAP *tunica albuginea plication

The angle of penile curvature was from a minimum of 22 degrees to a maximum of 88 degrees, the length of the erect penis, respectively, 12.2–21.1 cm, and girth – 9.7–16.8 cm.

All cases of horizontal penile curvature among the enrolled patients were acquired. Analysis of the causes of the curvature (the study did not include patients with Peyronie’s disease) showed that in most cases they were idiopathic or arose because of various medical interventions.

Thus, the analysis of the main characteristics of patients in the comparison groups demonstrated their comparability (p > 0.05).

The effectiveness of the treatment was assessed according to several criteria: penile curvature no more than 10 degrees, IIEF5 ≥ 21 points, satisfaction with the treatment according to the patient’s subjective perception, no subjective sensation of penile shortening, no subjective sensation of changes in the thickness or shape of the penis. Table 3 presents the treatment results divided into two groups.

**Table 3:** Evaluation of treatment outcomes using the composite endpoints method

Parameter	Successful	Satisfactory
No curvature > 10 degrees	+	-
IIEF5 ≥ 21 points	+	-
Shortening of the penis, subjective	+	-
Changing the thickness or shape of the penis, subjective	+	-
Satisfaction with treatment	+	-

*IIEF5* International Index of Erectile Function

All surgeries in both groups were successful according to the endpoints criteria. In the early and late postoperative periods, there were no cases of mortality in the two groups. In the postoperative period, there were no complications of anesthesia or deterioration of the general somatic status. The need for artificial ventilation or respiratory support did not arise in any case in the comparison groups. Also, there were no cases of heart failure that required inotropic support. Table 4 presents the dynamics of changes in the parameters of the penile state and functional status before and after the surgery.

**Table 4:** Dynamics of the parameters of the state of the penis and functional status in the comparison groups before and after surgery

**Parameter. I group; CR (** ***n*** ** = 9)**	**Before**		**3 months after**	**Difference**		**Р**	
Length of erect penis, cm	16.7 (± 1.9)		16.1 (± 1.8)	-0.6 (± 0.07)		0.470	
Girth of the erect penis, cm	13.3 (± 1.6)		12.1 (± 1.4)	-1.2 (± 0.14)		0.119	
The angle of the penile curvature, degrees	48.8 (± 17.1)		1 (1;1)	-47.5 (± 15.9)		**< 0.001**	
IIEF-5, points	21 (21;22)		25 (25;25)	+4 (3;4)		**< 0.001**	
**Parameter. II group; TAP (** ***n*** ** = 13)**	**Initially**		**Finally**	**Difference**		**Р**	
Length of erect penis, cm	16.1 (± 2.2)		13.6 (± 1.8)	-2.4 (± 0.8)		**0.006**	
Girth of the erect penis, cm	12.8 (± 1.48)		12.4 (± 1.7)	-0.3 (± 0.05)		0.585	
The angle of the penile curvature, degrees	48.2 (± 14.9)		1 (1;1)	-47.4 (± 14.7)		**< 0.001**	
IIEF-5, points	21 (20;22)		25 (25;25)	+5 (3;5)		**< 0.001**	

To determine the value of P, the Student’s criterion was used (in groups of continuous data with a normal distribution of the trait; described as mean +/-SD;), chi-square criterion (in groups of nominal data; described as n (%)), nonparametric Mann-Whitney U-criterion (in groups of continuous data with a distribution of different from normal; denoted as median and 25–75 quartiles.)

*CR *corporal rotation, *TAP *tunica albuginea plication

*IIEF5* International Index of Erectile Function

Data analysis showed that after CR there is a slight shortening (on average 6 mm) and a decrease (on average of 12 mm) in the girth of the erect penis (p < 0,05). CR can successfully eliminate curvature and improve copulatory function (change in both parameters *p* < 0.001). TAP caused significant shortening (on average 24 mm, *p* = 0.006) and a slight decrease (on average 3 mm) in the girth of the erect penis. TAP can successfully eliminate the curvature and improve copulatory function (change in both parameters *p* < 0.001).

Table 5 shows the comparative characteristics of the indicators of the postoperative state of patients in the comparison groups.

**Table 5:** Comparison of sample parameters of treatment results

Indicator	Group I CR (*n* = 9)	Group II TAP (*n* = 13)	Р
Average duration of hospitalization, days	1 (1;1)	1 (1;1)	1.0
Postoperative condition of the penis:			
Shortening of the penis > 1 cm, n (%)	0 (0%)	12 (92.3%)	**0.009**
Reducing penile girth > 1 cm, n (%)	8 (88.8%)	0 (0%)	**0.003**
Changing the length of the penis, cm	-0.6 (±0.07)	-2.4 (±0.8)	**<0.001**
Changing of the girth of the penis, cm	-1.2 (±0.14)	-0.3 (±0.05)	**<0.001**
Post-operative IIEF5, scores	25 (25;25)	25 (25;25)	0.946
Feeling numb, n (%)			
Up to 3 months after surgery	1 (11.1%)	3 (23.0%)	0.547
More than 3 months after surgery	1 (11.1%)	2 (15.3%)	0.801
Infectious complications, n (%)	0 (0)	0 (0)	0
Surgical seam insolvency, n (%)	0 (0)	0 (0)	0
Postoperative hematoma, n (%)	1 (11.1%)	0 (0%)	0.243
Palpable induration, n (%)	0(0%)	6 (46.1%)	0.057
Subjective patient satisfaction, n (%)	9 (100%)	11(84.6%)	0.789
Subjective shortening of the penis, n (%)	0 (0%)	4 (30.7%)	0.113
Subjective reduction of girth or change of penile shape, n (%)	1 (11.1%)	0 (0%)	0.243

To determine the value of P, the Student’s criterion was used (in groups of continuous data with a normal distribution of the trait; described as mean +/-SD;), chi-square criterion (in groups of nominal data; described as n (%)), nonparametric Mann-Whitney U-criterion (in groups of continuous data with a distribution of different from normal; denoted as median and 25–75 quartiles.)

IIEF5 - International Index of Erectile Function

*CR* corporal rotation, *TAP *tunica albuginea plication

The results analysis demonstrated that there were no significant complications in the postoperative period. The only complication in the early postoperative period was the occurrence of a small subcutaneous hematoma in a patient of group I; evacuating the hematoma during bandaging and using tight bandaging of the penis for one day solved the problem.

On average, all patients stayed for one day in the hospital, which meets the protocol criteria for Fast-track surgery. There were no changes in the hospital stay with either treatment.

Comparative analysis of objective and subjective parameters (length and girth of the erect penis) showed a significant difference in the comparison groups. Thus, after CR, there is a relatively decrease in the girth of the penis (*p* < 0.05), and after TAP, there is a significant decrease in the length of the penis (*p* < 0.05).

Numbness of the glans penis was found in both comparison groups. This parameter in the short and long terms did not differ in the comparison groups (in both cases, *p* > 0.5). A palpable induration in the surgical area was detected only in the TAP group, with a no statistical difference (*p* = 0.057).

Subjective assessment of the treatment results, as well as the condition of the penis in the postoperative period, did not differ in both groups (*p* > 0.05 for all three comparisons).

All patients were followed up for at least three months after surgery. For group I, the average observation period was 682 days with a 95% CI of 217–617 days (maximum period of 1116 days). For group II, the average observation period was 815 days with a 95% CI of 281–647 days (maximum period of 1276 days).

The analysis of the comparative intergroup treatment efficacy based on the criteria established in the study showed good treatment results for 8 (88.8%) patients of group I and 9 (69.2%) patients in group II (*p* = 0.577), according to Table 6. Satisfactory for everyone else. There were no negative outcomes.

**Table 6:** Predictors of complications

Factor	Sign	Univariate analysis			Multivariate analysis	
		χ^2^	OR (95% CI)	Р	OR (95% CI)	Р
Subjective shortening of the erect penis. Multifactor logit-regression: χ^2^ = 13.63; р = 0.0035	Height	6.65	0.28(-0.002;0.57)	0.052	0.6(-256;22.07)	0.099
	Weight	13.99	0.49(-0.09;1.0)	0.101	-	**-**
	Penile curvature before	3.7	0.07 (-0.009:0.15)	0.083	-0.04(-0.25;0.1)	0.709
	Angle before surge^1^ry > 60	5.06	2.7(0.12;5.28)	**0.040**	6.8(-1.75;15.4)	0.119
	Length before > 15.5 cm	2.56	0.42(-0.11;0.96)	0.126	-	-
	Changing the length > 2 cm	16.72	8.9(-11.2;29.1)	0.384	-	-
	Girth before surgery	2.61	0.52(-0.14;1.19)	0.123	-	-
	IIEF5 before surgery	1.19	-0.40(-1.13;0.33)	0.283	-	-
	Allergy	2.82	-1.4(-3.2;0.3)	0.113	-	-
Subjective reduction of girth or shape of the erect penis.	Curvature angle after surgery	1.38	0.47(-0.38;1.33)	0.278	-	-
	Length after surgery > 16 cm	8.08	0.77(0.07;1.47)	**0.029**	-	-
	Change in length > 1cm	20.59	-8.4 (-18.1:1.15)	0.085	-	-
	Girth before surgery	1.04	0.27(-0.26;0.80)	0.318	-	-
Palpable induration	Height	5.93	0.22(-0.009;0.45)	0.060	-	-
	Weight	8.23	0.17(0.02;0.33)	**0.027**	-	-
	Horizontal curvature	2.02	0.03 (-0.02:0.09)	0.158	-	-
	Penile curvature before	1.06	-0.05(-0.1;0.001)	0.310	-	-
	Angle before surgery > 60	2.02	1.46(-0.56;3.5)	0.158	-	-
	Length before surgery	3.56	0.45(-0.05;0.96)	0.082	-	-
	Changing length	12.19	2.1(0.32;4.04)	**0.021**	-	-
	Shortening > 2 cm	6.37	2.7(0.28;5.13)	**0.029**	-	-
	Girth before surgery	3.65	0.57(-0.06;1.2)	0.079	-	-
	Girth after surgery	6.73	0.88(0.08;1.68)	**0.031**	-	-

Univariate and multivariate logistic regression analysis made it possible to determine the predictors of complications (Table 6). The table shows the results with Chi-squared test.

A simple (univariate) logistic regression analysis among 22 patients of both comparison groups, demonstrated the significance in the prognosis of complaints of penile shortening only by the preoperative curvature angle > 60 degrees in group II (OR 2.7; 95% CI 0.12; 5, 28; *p* = 0.040). Based on this ratio, the degree of influence of the predictor on the risk of shortening was calculated. Consequently, a previous penile curvature > 60 degrees increases the likelihood of complaints on penis shortening by 2.7.

Obtained results helped to construct a model for predicting the occurrence of complaints of penile shortening in multivariate regression analysis (selection from predictor factors with a significance level of *p* < 0.1). There were no significant predictors.

Logistic regression analysis of the risk of changes in girth or shape of the penis was similar. Penile length over 16 cm after surgery was a significant predictor (OR 0.77; 95% CI 0.07; 1.47; *p* = 0.029). For predictors of development of palpable penile induration, the following factors were significant: weight (OR 0.17; 95% CI 0.02; 0.33; *p* = 0.027), change in penile length (OR 2.1; 95% CI 0.32; 4.04; *p* = 0.021), shortening > 2 cm (OR 2.7; 95% CI 0.28; 5.13; *p* = 0.029) and postoperative girth (OR 0.88; 95% CI 0.08; 1.68; *p* = 0.031).

Multivariate analysis did not show reliable results (*p* > 0,05 for all tests).

It should be noted that both methods of treatment have zero operating and hospital mortality and a low incidence of postoperative complications. Both methods of treatment are highly effective and safe for penile curvature correction.

## Discussion

The average normal size of an erect penis is a length of 13.1 (± 1.66) cm and a circumference of 11.6 (± 1.10) cm [27]. Currently, the criterion for the effectiveness of surgical treatment is a relatively straight penis (bend less than 20 degrees, which does not prevent the copulatory act). A shortening of the penis of less than 1.5 cm is also a favorable outcome [5, 15]. In the presented study, achieved results indicate the effective and safe use of both methods for correcting penile curvature.

Typical complications of any of the curvature correction methods are the postoperative hematomas (6.5%), painful or unpleasant indurations in the plication or rotation areas (1.4–73%), changes in the sensitivity of the glans penis (0–37%), shortening of the penis (up to 80%), wound infection (1.4%) and some more rare complications [15, 16]. There can be a technical complication such as a reverse deformity/curvature of the penis in the postoperative period, caused by spontaneous rupture of the thread or untied knot [16, 28]. During the study period, there were no cases of reverse deformity, a single development of postoperative hematoma did not require reoperation and was eliminated conservatively. The probability of numbness of the glans penis was comparable in both comparison groups. Numbness resolved on its own over time, which also correlates with the data of other authors [8]. Plication revealed several cases of subjective complaints of the presence of seals, which, nevertheless, did not differ significantly for the comparison groups [29, 30]. However, the authors believe that this conclusion is not accurate and is due to the small sample size.

When comparing the results, a statistically significant difference in the parameters of the penis was noted. However, these changes did not affect the subjective perception of the results of treatment. In general, the data are consistent with the results of the Shaeer CR surgery [22] and various TAP modifications [11–13].

Logistic regression analysis revealed interesting and debatable results. There is no doubt about the reliability of the risk of significant shortening of the penis after plication in patients with a bend of more than 60 degrees. This conclusion is consistent with the works of other authors [5, 15]. We consider the influence of the length of the penis more than 16 cm on the risk of changes in the girth or shape of the penis after surgery, and the effect of weight on the risk of developing palpable indurations as doubtful since there is only an indirect logical connection. The influence of plication on the absolute value of the girth of the penis after surgery are natural. However, there is a need for further studies with a larger sample.

Several researchers [14, 16] do not report the linear dimensions of the penile shortening, relying on the subjective perception of patients in the postoperative period, which is a less reliable presentation of treatment results in comparison with a complex subjective and objective assessment. Meanwhile, the results with accurate linear data in some cases indicate the absence of a significant change in length after TAP procedures [31]. Such works raise questions, reporting a slight objective decrease in the postoperative length of the penis. On the contrary, some studies indicate a more significant decrease in length after TAP from 0 to 2.5-5 cm, depending on the initial curvature angle and the type of curvature (apparently, the ventral version provides a more significant shortening) with TAP procedures [5, 15, 32]. Analyzing the works presented in more detail, the authors concluded about a different method for calculating the length of an erect penis, which provides a significant difference in TAP results. Thus, most authors before the surgery measure the length of the penis using a straight metal ruler, which gives unreliable results for the curve of the midline of the penis [32]. In addition, the authors do not describe the endpoints of measurement (glans to the skin of the pubis, or glans to the symphysis), which has the potential to influence (with different measurement techniques in different patients or the same patient at different stages of the study) the results within the framework of even one study [33]. It means that there is a need for a more detailed presentation in scientific works of the applied measurement methods, as well as the use of more reliable measurement with a flexible tape, measuring thread, or in another similar way.

The partial mobilization of the urethra to release the ventral parts of the corpora cavernosa can presumably be the solution to the problem of reducing the girth of the penis after rotational operations. Thus, it can help to solve the problem of deformation of the corpus cavernosum during its rotation, but this problem requires a separate study. In this case, the efficiency of straightening directly depends on the length of the rotation. That is, for this surgery, the more significant the curvature, the greater the mobilization of the neurovascular bundle and the implementation of many retaining sutures.

Separately, the authors would like to note that a high subjective assessment of the results of treatment, even with the shortening of the penis, is probably due to a significant improvement in copulatory function, leveling other negative sensations. However, copulatory function was not evaluated in the study.

Thus, the results and their comparison with the data presented earlier in scientific publications were analyzed. Comparative analysis is difficult due to the small number of works on the problem of penile curvature without Peyronie’s disease, as well as on the search for predictors of a specific type of complications after performing TAP or CR. There is a need for further study in larger samples and a comparison of the obtained data with the results of other authors. A significant advantage of the work is an isolated analysis (no relapse, one team of surgeons, refusal to re-enroll in the study, etc.) analysis of the influence of various factors on the likelihood of postoperative complications. Limitations of the research: relatively small samples, the average postoperative follow-up period of less than five years, single-center study.

## Conclusion

Both methods are safe, effective, and bring minimal risks of complications. Both surgical techniques lead to a change in the geometry of the erect penis: they allow to eliminate the curvature but are accompanied by shortening (Plication is more pronounced than Rotation) or a decrease in grith (Rotation is more pronounced than Plication). Patients should be warned about the consequences, and the method of surgical technique should be discussed with the presentation of possible outcomes.

Based on the results, important criteria for the selection of patients are formulated: Plication techniques are not recommended for patients with pronounced curvature, or initial dissatisfaction with the size of the penis; Rotation techniques require the greater mobilization of the neurovascular bundle and greater rotation, the more pronounced the curvature, which introduces a certain limitation in their use.

Rotation techniques require further study to evaluate and improve outcomes. It is required to conduct a comprehensive study of the joint application of techniques in complex variants of curvature.

## Data Availability

Data will be available on request.

## Abbreviations

BDMP: Birth Defects Monitoring Program
CR: corpus cavernosum rotation
FTS: fast-track surgery treatment protocol
IIEF5: International Index of Erectile Function
ISMU: Irkutsk State Medical University
TAP: tunica albuginea plication
